# Breakfast Staple Types Affect Brain Gray Matter Volume and Cognitive Function in Healthy Children

**DOI:** 10.1371/journal.pone.0015213

**Published:** 2010-12-08

**Authors:** Yasuyuki Taki, Hiroshi Hashizume, Yuko Sassa, Hikaru Takeuchi, Michiko Asano, Kohei Asano, Ryuta Kawashima

**Affiliations:** 1 Division of Developmental Cognitive Neuroscience at the Institute of Development, Aging and Cancer, Tohoku University, Sendai, Japan; 2 Department of Functional Brain Imaging at the Institute of Development, Aging and Cancer, Tohoku University, Sendai, Japan; 3 Smart Ageing International Research Centre at the Institute of Development, Aging and Cancer, Tohoku University, Sendai, Japan; Biological Research Center of the Hungarian Academy of Sciences, Hungary

## Abstract

Childhood diet is important for brain development. Furthermore, the quality of breakfast is thought to affect the cognitive functioning of well-nourished children. To analyze the relationship among breakfast staple type, gray matter volume, and intelligence quotient (IQ) in 290 healthy children, we used magnetic resonance images and applied voxel-based morphometry. We divided subjects into rice, bread, and both groups according to their breakfast staple. We showed that the rice group had a significantly larger gray matter ratio (gray matter volume percentage divided by intracranial volume) and significantly larger regional gray matter volumes of several regions, including the left superior temporal gyrus. The bread group had significantly larger regional gray and white matter volumes of several regions, including the right frontoparietal region. The perceptual organization index (POI; IQ subcomponent) of the rice group was significantly higher than that of the bread group. All analyses were adjusted for age, gender, intracranial volume, socioeconomic status, average weekly frequency of having breakfast, and number of side dishes eaten for breakfast. Although several factors may have affected the results, one possible mechanism underlying the difference between the bread and the rice groups may be the difference in the glycemic index (GI) of these two substances; foods with a low GI are associated with less blood-glucose fluctuation than are those with a high GI. Our study suggests that breakfast staple type affects brain gray and white matter volumes and cognitive function in healthy children; therefore, a diet of optimal nutrition is important for brain maturation during childhood and adolescence.

## Introduction

Brain development continues throughout childhood and adolescence [1]. Thus, adequate diet during these periods is thought to affect brain development and cognitive function. Recent studies have shown that eating breakfast has an immediate positive effect on cognitive function in children [2]–[4]. Moreover, skipping breakfast affects problem solving [5], short-term memory [5], and attention [6] in children. Although other studies have shown that eating breakfast does not significantly affect verbal memory [7]–[9], a recent review suggested that eating breakfast was associated with several positive effects on the cognitive functioning of well-nourished children [10]. Several studies of school breakfast programs have also shown the positive long-term effects of eating breakfast on the cognitive performance of well-nourished children [11], [12]. Therefore, it is thought that having breakfast has both short- and long-term positive effects on the cognitive functioning of children. In addition, several studies have focused on the effects of the quality of typical breakfasts, such as their contributions of energy content and macronutrient composition, on the cognitive performance of healthy children. For example, high-quality breakfasts are associated with better scholastic performance in well-nourished children [13], [14]. In addition, other studies have focused on the contribution of glycemic properties to the correlation between the quality of breakfast and cognitive functioning [7], [15]. The glycemic index (GI) is one measure of glycemic properties [16]. It measures the rate at which digestion of a food increases and maintains blood-glucose levels. In general, high-GI foods include rapidly digested low-fiber high-carbohydrate foods, which lead to an initial sharp peak in blood-glucose levels, followed by an overall decrease in circulating blood-glucose levels after a 2-h period. Conversely, low-GI foods cause a lower blood-glucose peak and generally result in a more sustained blood-sugar response, resulting in less fluctuation in blood-glucose levels and a more stable and efficient glucose supply to the brain compared with high-GI foods. Although few studies have addressed the correlation between the GI of breakfast foods and cognitive functioning, a recent study has shown that a breakfast of low-GI foods has immediate positive effects on such cognitive functions as attention [17]. Taken together, these findings suggest that the quality of breakfast staples may affect the cognitive functioning of well-nourished children.

As for the correlation between brain structure and cognitive function in children, several studies have focused on intelligence quotient (IQ) as a measure of cognitive function. Those studies have shown a significant positive correlation between brain regional gray matter volume and IQ [18]–[21]. Several gray matter regions are significantly correlated with IQ, including the prefrontal cortex [21], orbitofrontal cortex [18], and cingulate gyrus [18], [19]. Because of the correlation between breakfast staple type and several cognitive functions, it is plausible that a correlation also exists between breakfast staple type and gray matter volume. Because childhood and adolescence are crucial periods for acquiring cognitive function, revealing the correlations among type of breakfast staple, gray matter volume, and cognitive function is an important issue during development. However, little is known about these relationships in children.

Therefore, the purpose of this study was to determine whether a correlation among breakfast staple type, brain gray matter volume, and IQ exists. In addition, we also analyzed the correlation between type of breakfast staples and brain white matter volume. These analyses were accomplished by acquiring brain magnetic resonance (MR) images, data related to daily breakfast habits, and intelligence quotient data from 290 healthy children aged 5–18 years. With regard to breakfast type, boiled white rice and white bread are the two major breakfast staples in Japan. Therefore, we divided subjects into rice, bread, and both groups by their breakfast staple. The rice group consisted of subjects who habitually ate boiled white rice for breakfast, and the bread group consisted of subjects who habitually ate white bread for breakfast. The both group consisted of subjects who habitually ate both white bread and boiled white rice for breakfast, but ate either of the staples at a breakfast. We compared global and regional gray and white matter volumes and IQ among rice, bread, and both groups by applying voxel-based morphometry (VBM) [22]. VBM is an established automated neuroimaging technique that enables the global analysis of brain structure without a priori identification of a region of interest. It is not biased toward any specific brain region and permits the identification of potential brain structure differences or abnormalities. All analyses included two operations: the main analysis involved comparisons among bread, rice, and both groups; and the secondary analysis involved comparisons between bread and rice groups to highlight inter-group differences in gray and white matter volumes and IQs. The GI of boiled white rice is lower than that of white bread [23]. Although several factors other than GI, such as additional factors related to health status and the quality of breakfast foods, are also presumably associated with the correlations among type of breakfast staple, brain gray matter volume, and IQ, we initially hypothesized that the gray matter volume and IQ of the rice group would be significantly higher than those of the bread group.

## Methods

### Ethics statement

In accordance with the Declaration of Helsinki (1991), written informed consent was obtained from each subject and his/her parent after a full explanation of the purposes and procedures of the study was provided. Approval for these experiments was obtained from the institutional review board of Tohoku University.

### Participants

All subjects were healthy Japanese children who were recruited in the following manner. First, we distributed 29,740 advertisements summarizing the study to various kindergartens, elementary schools, junior high schools, and high schools in Miyagi Prefecture, Japan. Then, 1,423 parents of interested subjects contacted us by mail. Next, we mailed both a child version and a parent version of detailed study information to those parents. Then, 776 parents and subjects who were willing to participate contacted us again by mail. Subjects who had any history of malignant tumors, head trauma with a loss of consciousness lasting more than five minutes, developmental disorders, epilepsy, psychiatric diseases, or claustrophobia were excluded through a preliminary telephone interview, a mail-in health questionnaire, and an oral interview. Of the remaining subjects, brain MR images were not collected from eight subjects due to claustrophobia (3 subjects) and tiredness (5 subjects). Thus, we collected brain MR images from 290 subjects (145 boys, 145 girls; age range: 5.6 to 18.4 years) in the order in which the notifications of their intention to participate in the project arrived by mail. Trained examiners assessed IQ by administering the Japanese version of the Wechsler adult intelligence scale (WAIS; version 3) [24] to subjects whose ages were equal to or above 16 years. For subjects younger than 16 years old, we applied the Japanese version of the Wechsler intelligence scale for children (WISC version 3) [25]. We calculated the full-scale IQ, verbal IQ (vIQ), and performance IQ (pIQ) for each subject from their WAIS/WISC scores. In addition, we also calculated four composite scores, including the Verbal Comprehension Index (VCI), Perceptual Organization Index (POI), Processing Speed Index (PSI), and Working Memory Index (WMI) [24], [25]. We also collected information about the breakfast habits of each subject using mail-in questionnaires. We collected data from each subject in detail (if the subject was less than 10 years old, his/her parent answered all questions) as follows: “How many times per week does he/she eat breakfast?”; “Which main dish does he/she eat for breakfast: rice, bread, or both?”; “Which side dish(es) does he/she usually eat with the main dish: dairy products, vegetables, eggs, meat, fish or soybeans, soup of any kind, fruit, bean paste soup, fruit juice, black tea or coffee, or Japanese tea?”; and “How many side dishes does he/she usually eat with the main dish?”. We also collected data regarding the family's socioeconomic status from each subject's parent(s) by collecting family annual income information. Annual income data were collected using discrete variables as follows: annual income below 20,000 US dollars (if the currency exchange rate sets one US dollar as equal to one hundred yen), 1; 20,000–40,000 US dollars, 2; 40,000–60,000 US dollars, 3; 60,000–80,000 US dollars, 4; 80,000–100,000 US dollars, 5; 100,000–120,000 US dollars, 6; ≥120,000 US dollars, 7. Next, we divided subjects into rice, bread, and both groups by their breakfast staple. The characteristics of each group are shown in Table 1.

**Table 1:** Characteristics of the Rice Group, the Both Group, and the Bread Group.

	Rice group (*n* = 152)	Both group (*n* = 87)	Bread group (*n* = 51)	*P* a
Age [years], (mean ± SD, range)	11.4±3.34, 5.9–18.3	11.0±2.78, 5.7–17.2	11.3±3.09, 5.6–17.1	n.s.
Gender (no. of male: female)	74 ∶ 78	46 ∶ 41	25 ∶ 26	n.s.
Socioeconomic status^e^ (mean, range)	4.11, 1–7	3.59, 1–7	4.03, 2–7	<0.05^b^
Number of side dishes (mean, range)	3.74, 0–9	4.24, 0–8	2.71, 0–7	<0.01^c^, <0.001^d^

Rice group: Subjects who habitually ate boiled white rice for breakfast.

Both group: Subjects who habitually ate both boiled white rice and white bread for breakfast.

Bread group: Subjects who habitually ate white bread for breakfast.

a : Significance levels were evaluated with the Scheffe test if the analysis of variance was significant.

b : Comparison between the rice group and the both group.

c : Comparison between the rice group and the bread group.

d : Comparison between the both group and the bread group.

e Socioeconomic status is divided as follows; annual income below 2 million yen (∼20,000 US dollars), 1; 2–4 million yen, 2; 4–6 million yen, 3; 6–8 million yen, 4; 8 –10 million yen, 5; 10–12 million yen, 6; ≥12 million yen, 7.

### Image acquisition

All images were collected using a 3-T Philips Intera Achieva scanner. Using a Magnetization Prepared Rapid Gradient Echo (MPRAGE) sequence, three-dimensional high-resolution T1-weighted structural images (240×240 matrix, TR = 6.5 ms, TE = 3 ms, FOV = 24 cm, 162 slices, 1.0 mm slice thickness, voxel size = 1×1×1 mm) were collected.

### Image analysis of global gray and white matter volumes

After image acquisition, all T1-weighted MR images were analyzed using the Statistical Parametric Mapping 2 (SPM2) software [26] in MATLAB (MathWorks, Natick, MA). First, T1-weighted MR images were transformed into the same stereotactic space [27] by registering each of the images to the same template image. The template image used was derived from all subjects in this study. Then, tissue segmentation from the raw images to the gray matter, white matter, cerebrospinal fluid (CSF) space, and non-brain tissue segments was performed using the SPM2 default segmentation procedure. The voxel values of each segmented image consisted not of binary (i.e., 0 or 1), but of 256-grade (i.e., between 0/255 and 255/255) signal intensities according to their tissue probability. The linear normalized, segmented images were restored to the native space to determine the volumes of each segment. The actual volumes of the entire normalized, segmented, and restored segmented images were determined by summing voxel volumes (1 mm^3^) multiplied by each voxel value and divided by 255. Next, we determined the gray matter ratio (GMR) as the percentage of the gray matter volume divided by the intracranial volume. Intracranial volume was calculated by adding gray matter volume, white matter volume, and CSF space volume. White matter ratio (WMR) was calculated in the same manner.

### Image analysis of regional gray and white matter volumes

All T1-weighted MR images were analyzed using SPM2 in Matlab. First, the T1-weighted whole brain structural MR images from each subject were transformed into the same stereotactic space by the method described for the image analysis of global gray matter volume. Then, tissue segmentation from the transformed images to the gray matter, white matter, CSF space, and non-brain was performed using the SPM2 default segmentation procedure. Next, the segmented gray matter images were non-linearly normalized to the gray matter template described above using 7×8×7 non-linear basis functions in three orthogonal directions. These normalization parameters were reapplied to the T1-weighted whole-brain structural images of each subject to perform optimal spatial normalization. The optimally normalized T1-weighted images were segmented into gray matter, white matter, and CSF space. The normalized, segmented gray and white matter images were then modulated by calculating the Jacobian determinants, derived from the special normalization step, and multiplying each voxel by the relative change in volume, as in the method of Good *et al.* (2001) [28]. This modulation step was performed to correct for volume change in the non-linear normalization. The normalized, segmented, and modulated gray and white matter images were smoothed by convoluting a 12-mm-FWHM isotropic Gaussian kernel. This smoothing step was used to remove individual variations in gyral anatomy and to render data more normally distributed by the central limit theorem.

### Statistical analyses

We consistently treated the three-group comparison as the main analysis and the two-group comparison as an additional analysis. The three-group comparisons involved analyses including the bread, rice, and both groups. The two-group comparisons involved analyses including the bread and rice groups. In terms of the analyses of global gray and white matter volumes, to illustrate the differences in GMRs and WMRs among the rice group, the both group, and the bread group, we used analysis of variance (ANOVA) to compare the GMRs and WMRs of the groups in the three-group comparison. We also performed analysis of covariance (ANCOVA) adjusted for age, gender, socioeconomic status, average weekly frequency of eating breakfast, and the number of side dishes included with breakfast in the two-group analysis. The significance level was set at *P*<0.05. For analysis of the regional gray and white matter volume, we performed statistical analysis using SPM5 and VBM5 software (http://dbm.neuro.uni-jena.de/vbm/), an extension of SPM5. To illustrate the differences in regional gray and white matter volumes in three-group and two-group comparisons, we used the full factorial model in SPM5. In the analysis, age, gender, socioeconomic status, average weekly frequency of eating breakfast, and the number of side dishes included with breakfast were used as covariates. The level of statistical significance was set at *P*<0.05, corrected at the non-isotropic adjusted cluster level [29], with an underlying voxel level of *P*<0.005. Non-isotropic adjusted cluster-size tests can and should be applied when cluster-size tests are applied to data known to be non-stationary (i.e., not uniformly smooth), such as VBM data [29]. We used ANOVA to compare the scores obtained by members of the three groups in the full-scale IQ, verbal IQ, performance IQ, VCI, POI, PSI, and WMI on the WAIS/WISC (Version 3); we also performed ANCOVA to compare these factors adjusted for age, gender, socioeconomic status, average weekly frequency of eating breakfast, and number of side dishes included with breakfast in the two-group comparison. The significance level was set at *P*<0.05.

## Results

**Figure 1:**
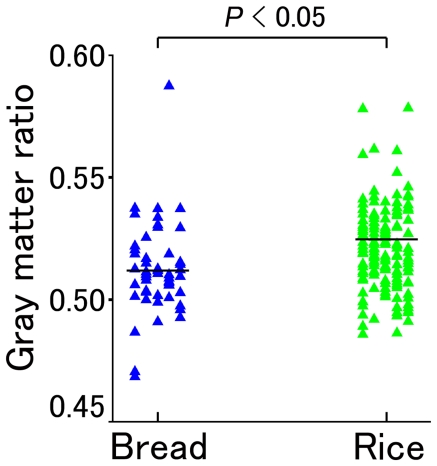
Gray matter ratio of the rice group and the bread group. The horizontal bars indicate gray matter mean ratio value in each group.

According to the ANOVA of global gray matter volume in the three group-comparison, the GMR of the rice group was significantly higher than that of the bread group (*t* = −3.21, *P* = 0.002, and *P*<0.01 using Scheffe's test) as shown in Figure 1 and Table 2. Additionally, the GMR of the rice group was significantly higher than that of the bread group according to the ANCOVA, after adjusting for age, gender, socioeconomic status, average weekly frequency of having breakfast, and number of side dishes eaten for breakfast in the two-group comparison (*t* = 3.250, *P* = 0.001).

**Table 2:** GMR and Intelligence Quotient (IQ) of the Rice Group, the Both Group, and the Bread Group.

	Rice group		Both group		Bread group		*P* a
	(*n* = 152)		(*n* = 87)		(*n* = 51)		
	Mean (SD)	Range	Mean (SD)	Range	Mean (SD)	Range	
GMR	0.521 (0.017)	0.486–0.578	0.516 (0.018)	0.477–0.580	0.512 (0.018)	0.469–0.587	<0.01^b^
WMR	0.290 (0.012)	0.257–0.315	0.290 (0.012)	0.260–0.323	0.293 (0.012)	0.264–0.331	n.s.
Full-scale IQ	103.7 (12.52)	77–136	102.0 (11.90)	77–137	99.9 (12.21)	71–129	n.s.
Verbal IQPerformance IQ	104.7 (13.93)102.1 (12.25)	76–14373–136	103.7 (13.93)99.5 (12.64)	67–15262–134	100.3 (11.91)99.6 (14.32)	67–12971–132	n.s.n.s.
VCI	104.6 (14.75)	73–145	103.2 (13.82)	64–151	100.3 (11.75)	65–126	n.s.
POI	102.3 (13.71)	68–146	100.3 (12.72)	69–131	97.9 (14.30)	74–134	n.s.
WMI	99.3 (14.16)	69–138	100.4 (11.66)	81–135	98.9 (12.64)	71–121	n.s.
PSI	103.1 (12.58)	72–136	100.4 (12.96)	66–136	104.8 (14.95)	69–133	n.s.

a : Significance levels were evaluated with the Scheffe test when the analysis of variance was significant.

b : Comparison between the rice group and the bread group.

n.s.: not significant.

GMR: gray matter ratio.

WMR: white matter ratio.

VCI: Verbal Comprehension Index.

POI: Perceptual Organization Index.

WMI: Working Memory Index.

PSI: Processing Speed Index.

The global white matter volume analysis using ANOVA for the three-group comparison revealed no significant differences in the WMRs among the three groups (*F* = 1.66, *P* = 0.193), as shown in Figure 1 and Table 2. However, the WMR of the bread group was significantly higher than that of the rice group after adjusting for age, gender, socioeconomic status, average weekly frequency of having breakfast, and number of side dishes eaten for breakfast according to the ANCOVA in the two-group comparison (*t* = 2.390, *P* = 0.018).

Next, we examined the possibility of a group-by-age interaction involving the GMR and WMR of the bread and rice groups. Because the mean age of all subjects in this study was 11.2 years old, we divided subjects into younger (under 11.2 years) and older (over 11.2 years) groups and analyzed the group-by-age interaction of GMR and WMR among the younger rice group, the younger bread group, the older rice group, and the older bread group. The mean and *SD* for age in each group was as follows: younger rice group, 8.65, 1.411; younger bread group, 8.81, 1.58; older rice group, 14.41, 1.85; and older bread group, 14.05, 1.68. We found no significant difference between the younger bread and younger rice groups (*t* = −0.46, *P* = 0.643) or between the older rice and older bread groups (*t* = −0.87, *P* = 0.386). The mean and *SD* of the GMR in each group was as follows: younger rice group, 0.524, 0.017; younger bread group 0.517, 0.018; older rice group, 0.517, 0.017; and older bread group, 0.506, 0.017. The mean and *SD* of the WMR in each group was as follows: younger rice group, 0.286, 0.011; younger bread group, 0.289, 0.008; older rice group, 0.295, 0.010; and older bread group, 0.299, 0.013. We used ANOVA to examine the possibility that the GMRs and WMRs of the four groups would differ significantly. Although we found no significant difference between the GMR of the younger rice group and that of the younger bread group (*t* = −1.86, *P* = 0.066), the GMR of the older rice group was significantly higher than that of the older bread group (*t* = −2.85, *P* = 0.005, and *P*<0.05 using Scheffe's test). We found no significant differences between the younger rice group and the younger bread group (*t* = 1.23, *P* = 0.225) or between the older rice group and the older bread group (*t* = 1.53, *P* = 0.131) in terms of WMR.

**Figure 2:**
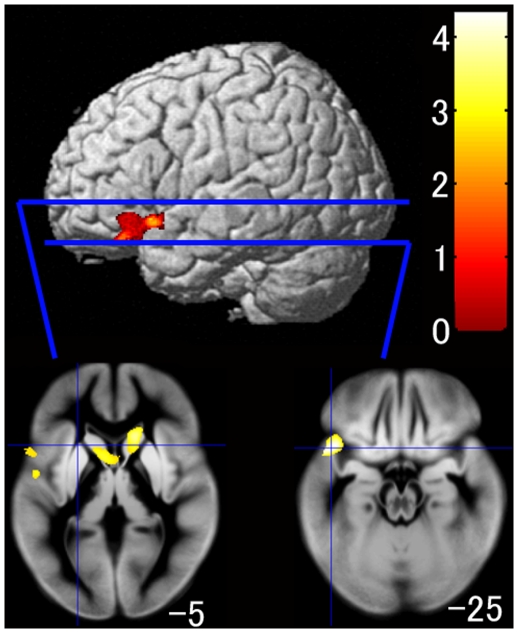
Gray matter regions with significantly larger volumes in the rice group as compared with the bread group after adjusting for age, gender, socioeconomic status, average weekly frequency of eating breakfast, and number of side dishes eaten for breakfast. Significant regions are superimposed onto a brain surface of a SPM template brain, as well as onto axial images of the mean normalized gray matter segments derived from all the subjects. The left side of the image represents the left side of the brain. Color scales indicate the t-score. The number at the bottom of the right side of each image indicates the value of the z-axis in the Talairach stereotaxic space.

**Figure 3:**
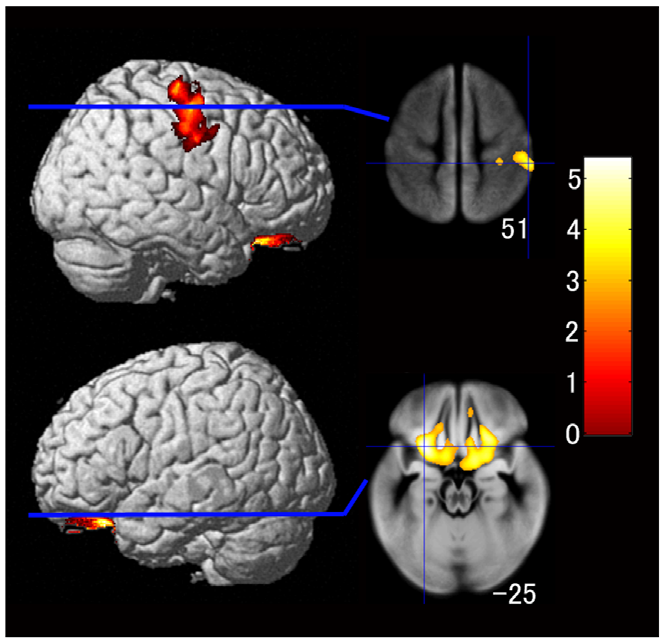
Gray matter regions with significantly larger volumes in the bread group as compared with in the rice group after adjusting for age, gender, socioeconomic status, average weekly frequency of having breakfast, and number of side dishes eaten for breakfast. Details are the same as in Figure 2.

Although the three groups did not differ significantly according to the regional gray matter volume analysis, the rice group had significantly larger regional gray matter volumes in an anatomical cluster in a gray matter region that included the left superior temporal gyrus and left inferior frontal gyrus (x, y, z = −48, 32, −25; *t* = 4.33, *P*-value of non-stationarity adjusted cluster level (*P*_corrected_)  = 0.043; *P*-value of family-wise error correction (*P*_FWE-corr_)  = 0.528), and the bilateral caudate nuclei (left; x, y, z = −25, 14, 15; *t* = 3.90; *P*_corrected_  = 0.013; *P*_FWE-corr_  = 0.954; right; x, y, z = 24, 20, 14; *t* = 4.13; *P*_corrected_ <0.001; *P*_FWE-corr_  = 0.764) after adjusting for age, gender, intracranial volume, socioeconomic status, average frequency of eating breakfast, and number of side dishes eaten for breakfast according to the two-group comparison, as shown in Figure 2. Conversely, the bread group had significantly larger regional gray matter volumes in an anatomical cluster in a gray matter region of the bilateral orbitofrontal gyri (left; x, y, z = −27, 35, −25; *t* = 5.38; *P*_corrected_  = 0.003; *P*_FWE-corr_  = 0.012; right; x, y, z = 26, 41, −27; *t* = 4.75; *P*_corrected_ <0.001; *P*_FWE-corr_  = 0.150) and the regions including the right precentral gyrus and the postcentral gyrus (x, y, z = 57, −15, 51; *t* = 4.30; *P*_corrected_ <0.001; *P*_FWE-corr_  = 0.426) after adjusting for age, gender, intracranial volume, socioeconomic status, average frequency of eating breakfast, and number of side dishes eaten for breakfast in the two-group comparison, as shown in Figure 3.

**Figure 4:**
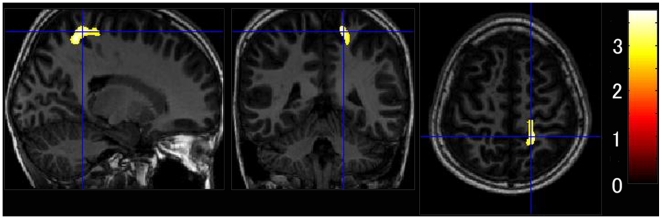
White matter regions with significantly larger volumes in the bread vs. the rice group after adjusting for age, gender, socioeconomic status, average weekly frequency of having breakfast, and number of side dishes eaten for breakfast. Color scales indicate *t*-scores.

Although the three groups did not differ significantly according to the regional white matter volume analysis, the bread group had significantly larger regional gray matter volumes in an anatomical cluster in a gray matter region that included the right precentral gyrus and the postcentral gyrus (x, y, z = 18, −45, 65; *t* = 3.77, *P*_corrected_  = 0.026; *P*_FWE-corr_  = 0.822) after adjusting for age, gender, intracranial volume, socioeconomic status, average frequency of eating breakfast, and number of side dishes eaten for breakfast in the two-group comparisons, as shown in Figure 4. No white matter regions were larger in the rice than in the bread group.

Although analysis of the relationship between IQ and breakfast staples showed no significant difference among the three groups in the three-group comparisons, as shown in Table 2, the full-scale IQ of the rice group was substantially higher than that of the bread group (*t* = 1.892, *p* = 0.06) after adjusting for age, gender, socioeconomic status, average frequency of eating breakfast, and number of side dishes in the two-group comparison. Neither vIQ nor pIQ was significantly different between the rice and bread groups (vIQ, *t* = 1.702, *p* = 0.090; pIQ, *t* = 1.621, *p* = 0.107). With respect to the four composite scores of the full-scale IQ, POI was significantly higher in the rice group than in the bread group (*t* = 2.333, *p* = 0.021) after adjusting for age, gender, socioeconomic status, average frequency of eating breakfast, and number of side dishes in the two-group comparison. No significant difference in SCI (*t* = 1.689, *p* = 0.093), WMI (*t* = 0.285, *p* = 0.776) or PSI (*t* = 0.308, *p* = 0.759) was found between the two groups.

## Discussion

To our knowledge, this is the first study to reveal the relationships among breakfast staple type, brain gray matter volume, and IQ by using brain magnetic resonance (MR) images in a wide age range of healthy children. Specifically, this study provided four novel findings. First, as hypothesized, the GMR of the rice group was significantly larger than those of the bread and both groups according to the global gray matter volume analysis in the three-group comparison. Additionally, the GMR of the older rice group was significantly higher than that of the older bread group, whereas we found no significant difference between the GMR of the younger rice and the younger bread groups. Second, the regional gray matter volume analysis showed that the rice group had significantly larger regional gray matter volumes in the left superior temporal gyrus and the bilateral caudate nuclei, whereas the bread group had significantly larger regional gray matter volume in the bilateral orbitofrontal gyri and the right precentral gyrus in the two-group comparison. Third, the WMR of the bread group was significantly larger than that of the rice group in the two-group comparison, and the regional white matter volume analysis showed that the bread group had significantly larger regional white matter volumes in the right precentral gyrus and the postcentral gyrus than did the rice group. Fourth, full-scale IQ and the POI scores were both substantially and significantly higher in the rice group compared with the bread group in the two-group comparison.

We showed that GMR was significantly higher in the rice group than in the bread group in the global gray matter volume analysis in the three-group comparison. In addition, we also showed that the rice group had larger regional gray matter volumes of the left inferior frontal gyrus and the bilateral caudate nuclei in the regional gray matter volume analysis of the two-group comparison. Moreover, the GMRs of the older rice group were significantly higher than those of the older bread group, whereas we found no significant difference between the younger rice and the younger bread groups in terms of GMR. These results indicate that the rice group had larger global gray matter volume and larger regional gray matter volume in several areas compared with the bread group, and that dietary habits influence gray matter volume in the long term. Although we adjusted for age, gender, socioeconomic status, average weekly frequency of having breakfast, and number of side dishes eaten for breakfast, the significant difference in GMR among the three groups suggests that several factors such as energy content, glycemic properties, and macronutrient composition may be reflected in the results. Indeed, a recent study showed that the total amount of energy obtained from breakfast was slightly lower in Japanese younger adults who habitually ate white rice than in those who habitually ate bread [30]. Therefore, the total amount of energy in the two breakfast groups in this study can be interpreted as not significantly different. With respect to glycemic properties, both boiled white rice and white bread have a large amount of carbohydrates. Carbohydrates are an important macronutrient included in daily human calorie requirements and have a high metabolism rate in the human body. In addition, carbohydrates are the most important source of brain energy, as they provide a large amount of blood glucose. A main difference between the components of boiled white rice and white bread is in their respective GIs. The GI of Japanese boiled white rice is reported to be lower than that of white bread (boiled white rice: white bread  = 68 ∶ 100) [23]. After the intake of a low GI food, a relatively small rise in blood glucose occurs, followed by a stable blood-glucose concentration [31], suggesting that low-GI foods provide a more stable and efficient glucose supply than do high-GI foods. Because a major portion of the glucose consumed by the brain is used for the maintenance of resting membrane potential in neurons [32], stable and efficient glucose supply is important for neurons. In addition, cerebral metabolic rates of glucose utilization are approximately two times higher in children compared with adults [33] because the mean number of synapses per neuron increases in preadolescence [34], [35]. Therefore, an efficient supply of glucose is important for brain maturation in children. The fat content of the two staples should be considered in examinations of macronutrient composition. Given the same amount of carbohydrates, white bread has a higher fat content than does boiled white rice [36]. In addition, the total amount of fat in breakfast is slightly higher in Japanese young adults who habitually eat white bread than in those who habitually eat boiled white rice [30]. Importantly, a high-fat diet reduces the expression of brain-derived neurotrophic factor [37], which plays a critical role in the synaptic activity and plasticity of mature neurons [38], [39]. Thus, one of the possible reasons that the GMRs of the rice group were significantly higher than those of the bread group concerns differences in the GI and fat content of boiled white rice and white bread.

The regional gray matter volume analysis showed that the rice group had significantly larger regional gray matter volumes in the left superior temporal gyrus and the bilateral caudate nuclei, whereas the bread group showed significantly larger regional gray matter volumes in the bilateral orbitofrontal gyri and the right precentral gyrus in the two-group comparison. Although we do not have a clear explanation for the regional gray matter volume differences between the two groups, a recent study showed that a postnatal diet high in nutrients is associated with larger caudate volume and higher vIQ in adolescents [40]. That study suggested that nutrition quality has long-term effects on regional gray matter volume in the caudate nucleus, as well as on cognitive function [40]. It may be that the rice group had larger regional gray matter volume in the bilateral caudate nuclei due to a more efficient supply of glucose compared with the bread group. However, further studies focusing on the correlation between brain function and several kinds of nutrition are needed to explain the mechanisms underlying the finding that the bread group had a larger regional gray matter volume in the right precentral gyrus.

We also demonstrated that the rice group had substantially higher full-scale IQ scores and significantly higher POI compared with the bread group in the two-group comparison. Recent studies have suggested that vIQ, but not pIQ, is particularly influenced by nutrition [40]–[42]. Although we did not find significant differences in vIQ between the rice and bread groups in this study, the POI (vIQ subcomponent) of the rice group was significantly higher than that of the bread group in the two-group comparison. Therefore, our results are partly consistent with recent studies. One of the above-mentioned studies showed that the volume of the caudate nucleus was significantly correlated with vIQ [40]. Moreover, a recent lesion-mapping study showed that the right caudate nucleus is related to POI [43]. Because caudate nucleus regional gray matter volume was larger in the rice group than in the bread group, the higher POI of the rice group may be explained by regional gray matter volume differences between the two groups.

Several discrepancies between the results of the three-group and those of the two-group comparisons emerged in this study. These inconsistencies may be derived from the lack of homogeneity in the subjects in the both group given that we placed subjects into the both group if they habitually ate both white bread and boiled white rice as a staple for breakfast, irrespective of frequency. Therefore, some subjects may have primarily eaten white boiled rice, whereas other subjects may have primarily eaten white bread for breakfast. For these reasons, the frequency with which participants in the both group ate white boiled rice or white bread may have differed. However, our results suggest that GMR tended to increase as a function of more frequent consumption of boiled white rice, as shown in Figure 1. Furthermore, full-scale and verbal IQs tended to increase as a function of eating boiled white rice, as shown in Table 2. Therefore, we propose that both two-group and three-group comparisons are important for revealing the correlations among type of breakfast staple, brain gray, and white matter volumes, and IQ.

This study had several limitations. First, this was a cross-sectional study. Therefore, we have shown a relationship between breakfast staple type, gray matter volume, and IQ, but we cannot identify the causal factor. Longitudinal studies are needed to clarify the causal factor. Second, to clarify the direct relationship between the type of the breakfast staple, gray matter volume, and IQ, we adjusted for age, gender, socioeconomic status, average weekly frequency of having breakfast, and number of side dishes eaten for breakfast. However, we cannot rule out other environmental confounding factors that might affect the correlations among breakfast staple type, gray matter volume, and IQ. Therefore, we cannot rule out the possibility that the correlation among breakfast staple, gray matter volume, and IQ may reflect more complex underlying mechanisms. With regard to the differences between white rice and white bread, we focused on GI differences; however, it is possible that other nutritional factors such as unsaturated fatty acids and vitamin content are also significantly different between rice and bread breakfast staples. Although the present study focused on the long-term effects of breakfast staple on gray matter and IQ and it is therefore difficult to control all other nutritional factors, the results of the present study should be interpreted with caution. Additionally, the number of subjects in each group was unequal because white boiled rice is a more popular breakfast staple in Japan than is white bread. Therefore, we cannot rule out the possibility that the unequal numbers of subjects in the groups may have biased the statistical analyses. Although the signal intensity of the gray matter segment is regarded as a volumetric measure of the gray matter, several factors such as white matter myelination also affect intensity [44]. In addition, we cannot rule out the possibility that the differences in breakfast staples modify the actual T1 signals in the brain MRIs, resulting in changes in the gray matter/white matter interface definition.

In conclusion, we have analyzed the relationships among breakfast staple type, gray matter volume, and IQ in 290 healthy children. We showed that the GMR of the rice group was significantly larger than that of the bread group using global gray matter volume analysis. In this analysis, the rice group was shown to have significantly larger regional gray matter volume in several regions, including the left superior temporal gyrus, and the bread group was shown to have significantly larger regional gray matter volume in several regions, including the bilateral orbitofrontal gyri. The full-scale IQ and the POI of the rice group were substantially and significantly higher than those of the bread group. Our study suggests that breakfast staple type affects brain gray matter volume and cognitive function in healthy children; therefore, a diet of optimal nutrition is important for brain maturation during childhood and adolescence.
